# Hydrogel for Sustained Delivery of Therapeutic Agents

**DOI:** 10.3390/gels10110717

**Published:** 2024-11-07

**Authors:** Adina Magdalena Musuc, Magdalena Mititelu, Mariana Chelu

**Affiliations:** 1 Institute of Physical Chemistry—Ilie Murgulescu, Romanian Academy, 060021 Bucharest, Romania; mchelu@icf.ro; 2 Department of Clinical Laboratory and Food Safety, Faculty of Pharmacy, “Carol Davila” University of Medicine and Pharmacy, 020956 Bucharest, Romania; magdalena.mititelu@umfcd.ro

## 1. Introduction

**Figure 1:**
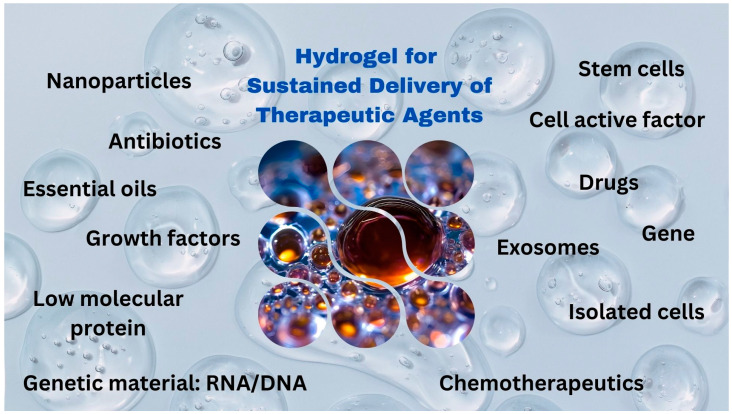
Various applications of hydrogels for sustained delivery of functional compounds.

In recent years, hydrogels have emerged as a highly promising platform for the sustained delivery of therapeutic agents, addressing critical challenges in drug delivery systems, from controlled release to biocompatibility. With their high-water content, biocompatibility, and tunable physical and chemical properties, hydrogels have enabled significant advancements in delivering a wide range of therapeutic agents, including small molecules, proteins, nucleic acids, and cells. This Special Issue, “Hydrogels for Sustained Delivery of Therapeutic Agents” of the journal *Gels* seeks to explore the latest innovations, challenges, and potential future directions in this field, highlighting the role of hydrogels in biomedicine (Figure 1).

Hydrogels are hydrophilic polymer networks that can absorb large amounts of water, resulting in a soft, tissue-like structure that can seamlessly interface with biological tissues. This unique feature allows hydrogels to deliver drugs in a more controlled manner than traditional drug delivery systems. By manipulating their molecular composition and structure, researchers have developed hydrogels with fine-tuned release kinetics, enabling the sustained delivery of therapeutic agents over extended periods. Furthermore, hydrogels can be engineered to respond to specific environmental cues—such as pH, temperature, or enzymes—making them ideal for targeted therapies in diseases such as cancer, cardiovascular diseases, and chronic inflammatory conditions.

This Special Issue aims to showcase cutting-edge research on the synthesis, characterization, and functionalization of hydrogels tailored for drug delivery applications. Novel hydrogel systems are increasingly designed for the co-delivery of multiple therapeutic agents, such as antibiotics with anti-inflammatory agents or chemotherapeutic drugs with immunomodulators. This strategy enables enhanced therapeutic efficacy while reducing side effects by maintaining localized, sustained release. Featuring a collection of ten papers, including six original research articles and four reviews, this Special Issue highlights the versatility of hydrogels in sustained drug delivery and explores how these systems are tailored for specific therapeutic challenges.

## 2. Contributions

The first research article published in this Special Issue is a study conducted by Pérez-González et al. [1]. This article focuses on caspofungin, an echinocandin-class antifungal used to treat severe and invasive fungal infections, and explores its application in treating cutaneous candidiasis—a challenging fungal skin infection. The study investigates the effect of two permeation enhancers, Azone and Transcutol-P, on caspofungin-loaded gels, evaluating the resulting formulations for their drug release profile, skin permeation, tolerability, and antimicrobial efficacy. In the treatment of fungal skin infections such as candidiasis, a significant challenge is achieving adequate drug retention and absorption in the skin layers while minimizing systemic absorption, which could lead to unwanted side effects. Pérez-González et al. [1] address this by incorporating Azone in a caspofungin gel (CPF-AZ-gel) to assess its potential for improved cutaneous application, comparing it to a standard caspofungin gel without permeation enhancers (CPF-gel). The study reports promising outcomes with the CPF-AZ-gel, which demonstrated superior skin retention and controlled drug release compared to the promoter-free formulation. Through in vitro release studies and ex vivo permeation testing on human skin, the authors showed that the CPF-AZ-gel provided an enhanced release profile while confining caspofungin’s diffusion primarily within the targeted skin layers. Additionally, both formulations displayed pseudoplastic behavior, making them suitable for easy and uniform application, as well as excellent spreadability and compatibility with skin biomechanics. Notably, antimicrobial efficacy testing confirmed that both formulations were effective against *Candida glabrata*, *Candida parapsilosis*, and *Candida tropicalis*, while *Candida albicans* exhibited resistance. Histological analysis of skin samples confirmed that both gels were well tolerated, indicating their suitability for clinical use in patients with cutaneous candidiasis, particularly those who may not respond to or tolerate conventional antifungal therapies.

Petrini et al. explore [2] a novel application of photodynamic therapy (PDT) using an aminolevulinic acid-based gel in periodontal tissue repair. The study assesses how photodynamic therapy (ALAD-PDT) with red LED irradiation affects human gingival fibroblasts (hGFs) and osteoblasts (hOBs) cultured on porcine acellular dermal matrix membranes (PADMMs), a common material used in periodontal surgery. The findings offer promising insights into PDT’s potential for accelerating healing and reinforcing the stability of membrane grafts in oral surgery. In this study, human gingival fibroblasts and osteoblasts obtained from dental patients were cultured on PADMMs and subjected to three treatment groups: a control group (CTRL) without exposure, a group receiving red LED irradiation only, and a group treated with ALAD-PDT (45 min of aminolevulinic acid incubation followed by 7 min of red LED exposure). The results show that ALAD-PDT significantly enhanced cellular proliferation and organization, forming a dense network of cells on PADMMs. Further assays—including MTT, histology, SEM, and mineralization assays—confirmed that ALAD-PDT significantly increased collagen and fibronectin production in fibroblasts and promoted bone marker expression in osteoblasts, suggesting that ALAD-PDT facilitates faster and more robust healing outcomes.

Hussain et al. [3] explore a cutting-edge, non-invasive approach to delivering valproic acid (VA) directly to the brain via nasal administration. This innovative study leverages computational predictive modeling and nanoemulsion gel formulation to overcome the challenges of conventional valproic acid delivery routes, which often result in systemic side effects, rapid hepatic metabolism, and low bioavailability in the brain. The study incorporated GastroPlus Version 9.8.3 and HSPiP (Hansen Solubility Parameters in Practice) programs to identify optimal excipients, evaluate formulation parameters, and predict the in vivo performance of valproic acid. GastroPlus simulations provided insights into drug absorption, distribution, and predicted the advantages of nasal administration over traditional oral and parenteral routes. Hansen solubility parameters helped select excipients that offered optimal miscibility, forming stable and effective nanoemulsion gels.

The development of advanced hydrogel systems for wound care has significant potential in managing chronic conditions, especially for diabetic patients facing slow or non-healing wounds. The research article by Aldakheel and colleagues [4] showcases a promising approach that merges green synthesis techniques with hydrogel technology to address this urgent healthcare need. Diabetic chronic wounds are prone to infection, poor healing outcomes, and an increased risk of complications like limb amputation, making effective, rapid-healing wound care solutions critical. In this study, the authors developed a polysaccharide-based hydrogel infused with silver nanoparticles (AgNPs) synthesized through an eco-friendly process using garlic extract, which acts as a reducing agent. The hydrogel matrix, composed of chitosan, starch, and alginate (PsB), was further modified with acrylamide to improve its mechanical and adhesive properties. The green-synthesized AgNPs demonstrated effective antibacterial activity against *Klebsiella pneumoniae* and *Staphylococcus aureus*, two bacterial strains commonly associated with wound infections. The choice of silver, known for its broad-spectrum antibacterial properties, enhances the hydrogel’s functionality by providing infection control, while the polysaccharide matrix aids in creating a moist wound environment conducive to healing. By using green synthesis for AgNP production, this research aligns with the growing demand for sustainable medical materials and demonstrates a practical alternative to traditional, chemical-based wound care products.

In their paper, Ji and colleagues [5] present an innovative approach to anticancer drug delivery. This study addresses the significant challenge of safely delivering toxic anticancer agents, such as tamoxifen citrate, in a controlled and sustained manner to improve therapeutic efficacy and reduce side effects. Utilizing a modified coaxial electrospraying technique, the authors created microparticles composed of tamoxifen citrate (TC) within a matrix of ethylcellulose (EC), coated with stearic acid (SA) to regulate the drug’s release. The use of ethylcellulose as the core matrix provided a robust foundation for the encapsulation, while the stearic acid layer functioned as an additional control mechanism to moderate the diffusion of the drug. The authors analyzed the morphology, structural compatibility, and physical state of the microparticles using advanced characterization techniques, including scanning and transmission electron microscopy (SEM and TEM), X-ray diffraction (XRD), and Fourier-transform infrared spectroscopy (FTIR). These methods validated the integrity and uniformity of the SA coating, which was integral to the observed sustained release profile.

In another research paper, Slavkova and colleagues [6] present a promising therapeutic approach for pediatric atopic dermatitis, utilizing budesonide-loaded nanoparticles in a hydrogel matrix to enhance treatment precision and efficacy. Budesonide, a corticosteroid often used in treating skin inflammation, has shown limited success in topical applications due to side effects and challenges related to its stability and permeability in the skin. This study leverages the pH differences observed in atopic dermatitis lesions to create a responsive nanocarrier, Eudragit L100, which enhances budesonide’s release to the inflamed site. Nanoparticles were created via a nanoprecipitation method, producing particles with a mean size of 57 nm, a negative surface charge (−31.2 mV), and high drug encapsulation efficiency (~90%). Cytotoxicity assays on HaCaT keratinocyte cells indicated their safety for skin applications, making this a viable delivery method for young patients. The nanoparticles were subsequently incorporated into two types of hydrogels: methylcellulose or Pluronic F127, which were rigorously analyzed for characteristics like pH, occlusion, rheology, spreadability, and drug release profiles. These hydrogels demonstrated controlled, targeted release of budesonide, positioning them as an effective solution for treating atopic dermatitis in pediatric patients. This study underscores the potential of nanoparticle-infused hydrogels as advanced, localized treatment options for skin conditions, especially where traditional therapies may fall short.

In their comprehensive review, Chen et al. [7] examine recent innovations in hydrogel coatings applied to titanium and titanium alloy implants, materials widely used due to their mechanical properties and biocompatibility. However, titanium’s performance within the physiological environment can be limited, particularly in promoting cellular interactions and biological integration. To address these challenges, hydrogel coatings offer a biochemical approach to enhance surface bioactivity by attaching functional biomolecules, including proteins, growth factors, and peptides. This biochemical strategy enables the implant surface to better support cell adhesion, proliferation, and differentiation, which are critical for improved biocompatibility and long-term implant success. The review highlights both natural polymers (e.g., collagen, gelatin, chitosan, alginate) and synthetic polymers (e.g., polyvinyl alcohol, polyacrylamide, polyethylene glycol, polyacrylic acid) used in hydrogel coatings. Various application methods for creating these coatings, such as electrochemical deposition, sol–gel processes, and layer-by-layer assembly, are thoroughly reviewed. Furthermore, the authors explore the five key benefits hydrogel coatings bring to titanium implants: enhanced osseointegration, improved angiogenesis, the modulation of macrophage responses (promoting an anti-inflammatory effect), antimicrobial properties, and the capability of localized drug delivery.

The review of Chelu et al. [8] delves into the growing field of *Aloe vera*-based hydrogels, emphasizing their advantages as biocompatible, therapeutic wound dressings. *Aloe vera* is renowned for its healing properties, and when integrated into hydrogels, it provides an ideal environment for the promotion of tissue repair, mitigating inflammation, and delivering bioactive agents directly to wound sites. This review discusses the synthesis techniques and structural characteristics of these hydrogels, examining how their properties support wound healing. Chelu et al. [8] explore the various mechanisms through which therapeutic agents are released from *Aloe vera* hydrogels, including diffusion, swelling, and degradation, which allow for controlled and sustained drug delivery. In addition to enhancing wound closure, these hydrogels offer significant antimicrobial and anti-inflammatory benefits due to *Aloe vera*’s natural compounds and the potential for incorporating additional therapeutic agents. The review covers different approaches for embedding antimicrobial and anti-inflammatory agents into these hydrogels, thus expanding their efficacy against infections and inflammation.

Villa et al. [9] examine the use of natural deep eutectic solvents (NaDESs) as eco-friendly, effective solvents in the cosmetic and pharmaceutical industries. Recognized as a new generation of green solvents, NaDESs provide a safer, non-flammable alternative to conventional ionic liquids and can be tailored for both lipophilic and hydrophilic molecules. This versatility makes them ideal for various applications, from sustainable extraction processes to biocompatible drug delivery systems. In pharmaceuticals, NaDESs are highlighted for their role as biopolymer modifiers, where they act as “therapeutic deep eutectic systems”. These systems enhance the solubility and stability of active ingredients, offering potential improvements in drug delivery. In cosmetics, NaDESs show promise in forming more sustainable, efficient formulations, providing a means for the stable incorporation of bioactive ingredients in topical and dermal applications. This review synthesizes the current understanding of NaDES applications in these fields, discussing both their practical uses and the challenges ahead. By examining the multifunctionality of NaDESs, the review underscores their potential to transform formulation practices within cosmetics and pharmaceuticals, advocating for their broader adoption as a green solution in bioactive ingredient delivery.

Chelu [10] presents a comprehensive analysis of recent innovations combining essential oils with hydrogel technology for diverse applications such as biomedical, dental, cosmetic, food, packaging, and heritage restoration. This review explores the synthesis, polymeric sources, and cross-linking techniques used in these hydrogels, emphasizing their biocompatibility, non-toxicity, antibacterial properties, controlled release capabilities, and cytocompatibility. The unique properties of essential oils, including their bioactivity and aromatic potential, are examined alongside their extraction and encapsulation processes. The review delves into the benefits and challenges of these methods, addressing issues such as the volatility, solubility, and stability of essential oils within hydrogel matrices. The encapsulation of essential oils in hydrogels enhances both stability and biological efficacy, making these formulations viable for sustained release applications in health and cosmetic products as well as food preservation and cultural conservation. Chelu’s review outlines the challenges and limitations faced in essential oil hydrogel technologies and discusses their promising future, noting significant potential across a broad spectrum of fields due to the multifunctionality of hydrogels and the enhanced delivery of natural bioactive compounds.

## 3. Future Directions in Hydrogel-Based Therapeutics

While the studies presented in this Special Issue illustrate the tremendous potential of hydrogel systems, challenges remain that require collaborative research efforts. The field is moving towards more personalized, patient-specific approaches, leveraging advanced techniques such as 3D printing and machine learning to create hydrogels tailored to individual needs. The integration of “smart” responsive materials that adjust drug release based on real-time feedback holds promise for next-generation, self-regulating delivery systems. Furthermore, the inclusion of both therapeutic and diagnostic functionalities within a single hydrogel platform—often referred to as “theranostic” applications—is an exciting development that could transform treatment paradigms for chronic and complex diseases.

## 4. Conclusions

This Special Issue captures both the depth and diversity of hydrogel-based drug delivery research. It reflects the field’s journey from theoretical advancements to tangible applications and encourages continued exploration to overcome existing limitations. By integrating high-quality reviews and pioneering research, we aim to inspire innovation and collaboration among researchers and industry professionals working towards safer, more effective, and patient-centered drug delivery solutions. We hope that this collection will serve as a valuable resource for anyone interested in the field of hydrogels for sustained therapeutic delivery and look forward to witnessing the future impact of these advancements on modern medicine.

We would like to extend our deepest gratitude to the journal *Gels* for the invaluable opportunity to produce this Special Issue, and we express our heartfelt thanks to the editorial team for their tireless support, especially our managing editor Ms. Miranda Song, who provided continuous guidance throughout the submission and publication process. Finally, this Special Issue could not have been realized without the dedication of the contributing authors and the conscientiousness of our reviewers, whose critical insights and commitment have ensured the high quality and scientific rigor of this collection.
